# Correlation of Serum Lipid P rofile with Histological and Seminal Parameters of Testis in The Goat

**Published:** 2013-07-31

**Authors:** Ali Louei Monfared

**Affiliations:** Department of Basic Sciences, Faculty of Veterinary Para-Medicine, Ilam University, Ilam, Iran

**Keywords:** Lipid, Testis, Goat, Histology

## Abstract

**Background:**

The lipid composition of a mammal’s spermatozoa and seminal plasma vary in both structure and function. Evidence exists to suggest that dietary supplementation with the appropriate polyunsaturated fatty acids (PUFAs) affects spermatogenesis, semen quality and sperm motility. Therefore, this study has been conducted to evaluate the correlations between serum lipid profile and histological, anatomical and seminal parameters of testes in clinically healthy goats.

**Materials and Methods:**

In this analytic, cross-sectional study, we chose a total of ten mature Iranian male goats that comprised a homogenous group through simple random sampling. Blood samples were taken from the jugular vein; the sera were separated and subsequently used for measurement of serum lipids, lipoproteins and testosterone levels. In addition, we collected semen from the animals and evaluated the seminal characteristics. We also performed histological and anatomical assessments of the testes.

**Results:**

The findings demonstrated that serum levels of high density lipoprotein (HDL-c) had a significant positive correlation with interstitial testicular tissue area (r=0.73; p<0.001), seminiferous tubule area (r=0.61; p<0.01), the number of Leydig cells (r=0.53; p<0.05), the diameter of the Leydig cell nuclei (r=0.54; p<0.05), scrotal circumference (r=0.83; p<0.001), testis weight (r=0.72; p<0.001), the number of live, normal sperm (r=0.94 ; p<0.001) and serum testosterone levels (r=0.88; p<0.001). Significant but negative correlations were found between serum triglyceride concentration and seminiferous tubule area (r=-0.53; p<0.05), the diameter of the Leydig cell nuclei (r=-0.55; p<0.05), testis weight (r =-0.64; p<0.01), total sperm number (r=-0.82; p<0.001), number of live, normal sperm (r=-0.55; p<0.05) and serum testosterone levels (r=-0.79; p<0.001). In addition, a significant negative correlation was observed between serum very low density lipoprotein (VLDL-c) concentration and the percent of live sperm (r=-0.67; p<0.01), and serum testosterone levels (r=-0.65; p<0.01).

**Conclusion:**

The present results indicated that among serum lipids only the levels of HDL-c positively correlated with testicular parameters. High serum triglyceride levels exerted direct adverse effects at the testicular level, which was mainly observed in the seminiferous tubules (STs), characterization of Leydig cells and semen quality.

## Introduction

It is well known that the lipid composition of a mammal’s spermatozoa and seminal plasma differ in both structure and function. Evidence exists to suggest that dietary supplementation with appropriate polyunsaturated fatty acids (PUFAs) affects spermatogenesis, semen quality and sperm motility (1, 2). In addition, Esmaeili et al. (3) have reported that dietary fatty acid sources affect the PUFA composition of the ram´s spermatozoa.

Lipoproteins transfer various lipids such as cholesterol from the tissue of origin to target sites, where the lipid complex is delivered via lipoprotein receptor-mediated uptake. Also, the supply of the Steroids required for cellular activities, including membrane formation, steroid hormone secretion, and the post-translational modification of proteins is regulated by lipoproteins (4). Lipoprotein- derived cholesterol is a major source of substrate for steroidogenic tissues, including the Leydig cells of some species (5-8).

The ratios of triglycerides and cholesterol to a polipoprotein B in the plasma are inversely related to testosterone levels (9). Also, plasma testosterone levels are related to the serum lipid profile in normal and infertile men (10). It has been suggested that high serum levels of cholesterol and triglyceride are associated with poor semen quality (11). In another study, Sehastian et al. reported that infertility might be associated with the changed lipid metabolism in seminal plasma in male individuals (12). Seminal parameters have been shown to be negatively correlated with serum very low density lipoprotein (VLDL-c) and total triglyceride values (13). Hypercholesterolemia and hypertriglyceridemia in rabbits possibly produce a decreased sperm capacity in an acrosome reaction (14). Cholesterol-enriched diets in rabbits have adverse effects on leydig and sertoli cells secretary functions, initiation and maintenance of spermatogenesis, sperm cytoskeleton formation, the sperm maturation process in the epididymis, and sperm ability to initiate embryonic development (15).

Although goats are economically a very important domestic mammal, there are very few data in the literature concerning the testicular cell populations and endocrine secretion in this species (16). Because sperm lipids contain extremely high proportions of fatty acids (1) ,it is necessary to establish a link between serum lipid profile to both testicular structure and function. Accordingly, this study seeks to evaluate correlations between serum lipid profile and histological, anatomical and seminal parameters of testes in clinically healthy goats.

## Materials and Methods

### Experimental animals and design

In this analytic, cross-sectional study, a total of ten mature Iranian male goats that comprised a homogenous group (aged 28.7 ± 2.2 months and 43.7 ± 3.1 kg average body weight) were selected from local intensively managed goat flocks (Ilam, Iran) through simple random sampling. The experiments were carried out from late May to July 2009, at the research farm of Ilam University. All animals were in good health and of proven fertility since they were being used as sires in a specialized goat farm. All goats received a daily diet of 702 g alfalfa hay, 69.9 g barley and 141 g of a commercial concentrate. The diet contained 2.4 Mcal ME/ kg, 12.1% crude protein, 1.1% calcium, and 0.31% phosphorus. All goats had free access to mineral blocks and fresh water. This unit was supervised by an experienced veterinarian and the prevention of internal and external parasites were a routine practice.

### Blood sampling and analysis

Blood samples (15 ml) were taken from the jugular vein between 09:00 and 10:00 am after overnight fasting and collected in vacutainers (Becton Dickinson Co., USA). Serum was separated by centrifugation at 2500 rpm for 20 minutes and stored at -20˚C until analysis. The sera were analyzed for total cholesterol and triglyceride levels using enzymatic kits (Pars Azmoon, Iran). Lipoproteins were isolated from sera by a combination of precipitation and ultracentrifugation. The precipitation method was used for measuring high density lipoprotein (HDL-c) particles. In this method, after addition of sodium phosphotungstate- magnesium to the serum, the non-HDL lipoproteins that sedimented by centrifugation (10000 g for 5 minutes) were aggregated. Then, the residual cholesterol was enzymatically measured (17, 18). Low density lipoprotein (LDL-c) was calculated as the difference between cholesterol measured in the precipitate and in the HDL-c fraction, while VLDL-c was estimated as one-fifth of the concentration of triglycerides (19). In addition, serum testosterone level was measured by a radioimmunoassay method that employed a diagnostic kit (Immunotech, SA, France, PI-1119).

### Semen collection and quality evaluation

Semen was collected in the morning by using an artificial vagina and transported to the laboratory (at 37˚C) within 10-15 minutes, then placed in a water bath at 37˚C. All ejaculates were evaluated for volume, sperm concentration (photometer, IMV, L’Aigle, France), percent of live sperm (eosin-nigrosin method), and percentage of normal sperm in the stained smear (20).

### Anatomical measurements

Scrotal circumference was measured using a tape-measure. The combined testis width, then testis length in the scrotum was determined by the use of a caliper (Mitutoyo, Kanagawa, Japan).

### Histomorphometrical assessment

The right testis from each animal was removed from the body under local anesthesia. Before surgery, all animals received intramuscular (IM) injections of 1 ml of Rompum (Bayer) per 100 kg of body weight. All surgical procedures were performed by a licensed veterinarian and the obtained testes were trimmed out from the scrotum and epididymis. Then, testes were weighed and cut along the longitudinal axis to obtain transverse sections of the seminiferous tubules (STs). Tissue samples, 1-3 mm thick, were taken near the tunica albuginea. Sections of 4 μm thickness were stained with hematoxylin and eosin (H & E) and viewed by a light microscope (Nikon Eclipse E800). Fields with sections of STs in circular transverse section were selected at 100x magnification; photographs were taken with a digital camera (Coolpix 950, Nikon, China) and stored. Histometric and cytometric studies were conducted using Image Tool® 3.0 software (UTHSCSA, San Antonio, USA). For this purpose, the boundary demarcation of STs was performed using the software and the area of the STs in each field was deducted from the total area of the field. Thus, the area of the STs and the area of the interstitial tissue were deduced. To measure the tubular diameter and the height of STs epithelium, at least thirty round or nearly round ST cross-sections were chosen at random and measured for each animal. In addition, the number of Leydig cells per 103 mm of the interstitial area was counted in five fields from each animal, at 1000x magnification. Finally, the diameters of the Leydig cell nuclei were measured according to the Faridha et al. (21) method.

### Statistical analysis

Results were presented as mean ± standard error of mean (SEM) for serum lipid profile, histological, anatomical and seminal parameters and the levels of serum testosterone. The data were statistically analyzed using the SPSS statistical software package, version 10.0.1 (SPSS Inc., Chicago, IL, USA. Prior to analysis, the Kolmogorov-Smirnova test was used to check normal distribution of the data. Then, to evaluate the correlation of the serum lipid profile with testicular cell populations and endocrine secretion, Pearson’s partial correlation coefficient analyses were conducted. A p value <0.05 was considered statistically significant.

## Results

Mean ± SEM and correlation coefficients between serum lipids and lipoproteins with histological measurements of testis in the Iranian male goat are presented in tables 1-3. In the present work, the level of serum HDL-c showed a positive, highly significant correlation with interstitial testicular tissue area (r=0.73; p<0.001) and STs area (r=0.61; p<0.01). There was a weak positive correlation of these attributes with the number of Leydig cells (r=0.53; p<0.05) and the nuclei diameters of the Leydig cells (r=0.54; p<0.05). In addition, significant but negative correlations were found between the level of triglycerides in the serum and STs area (r=-0.53; p<0.05) or diameter of the Leydig cell nuclei (r=-0.55; p<0.05).

**Table 1:** Mean ± standard error (SEM) of serum lipids and lipoproteins in the Iranian male goat (n=10)


Total cholesterol (mg/dl)	Total triglycerides (mg/dl)	Very low density lipoprotein (mg/dl)	Low density lipoprotein (LDL-c) (mg/dl)	High density lipoprotein (HDL-c) (mg/dl)

47.25 ± 2.9	46.6 ± 1.5	10.3 ± 2.8	17.8 ± 1.1	18.1 ± 0.8


**Table 2:** Mean ± standard error (SEM) of the histological measurements of testis in the Iranian male goat (n=10)


Interstitial testicular tissue area (%)	Area of seminiferous tubules (STs) (%)	Height of STs epithelium (μm)	STs diameter (μm)	Number of Leydig cells (×10^3^ mm area of the testis)	Diameter of Leydig cell nuclei (μm)

9.3 ± 1.3	88.8 ± 0.88	72.90 ± 1.9	215.7 ± 3.7	19. 5 ± 1.6	3.2 ± 0.1


**Table 3:** Correlation coefficients between serum lipids and lipopr oteins with histological measurements of testis in the Iranian male goat (n=10)


Serum lipid profile/ histological measurements of testes	Total cholesterol (mg/dl)	Total triglycerides (mg/dl)	Very low density lipoprotein (VLDL-c) (mg/dl)	Low density lipoprotein (LDL-c) (mg/dl)	High density lipoprotein (HDL-c) (mg/dl)

**Interstitial testicular tissue area (%)**	0.16	0.19	0.21	0.11	0.73***
**Seminiferous tubules (STs) area (%)**	0.23	-0.53*	0.18	0.18	0.61**
**STs epithelium height (μm)**	0.12	0.29	-0.21	0.15	0.18
**STs diameter (μm)**	0.11	0.13	0.25	0.12	0.15
**Number of Leydig cells (×10^3^ mm area of testis)**	0.15	0.24	0.31	-0.23	0.53*
**Diameter of the Leydig**	0.15	-0.55*	0.13	0.27	0.54*


*; Significant at p<0.05, **; Significant at p<0.01 and *** ; Significant at p<0.001.

The mean ± SEM of anatomical measurements of the testes, live body weight of the animals and their correlation coefficients with the serum lipid profile are presented in tables 4 and 5. Correlation coefficients of HDL-c with the scrotal circumference (r=0.83; p<0.001) and testis weight (r=0.72; p<0.001) were highly significant. There were significant negative correlations observed between total serum triglyceride level and testis weight (r=- 0.64; p<0.01).

**Table 4:** Mean ± standard error (SEM) of anatomical measurements of the testes and live body weight of the Iranian male goat (n=10)


Scrotal circumference (cm)	Testis width (cm)	Testis length (cm)	Testis weight (g)	Live body weight (kg)

42.23 ± 2.5	265.17 ± 5.3	7.38 ± 1.6	3.59 ± 1.2	29.61 ± 1.6


**Table 5:** Correlation coefficients between serum lipid profile with anatomical measurements of the testis and live body weight of Iranian male goat (n=10)


Serum lipid profile/ histological measurements of testes	Total cholesterol (mg/dl)	Total triglycerides (mg/dl)	Very low density lipoprotein (VLDL-c) (mg/dl)	Low density lipoprotein (LDL-c) (mg/dl)	High density lipoprotein (HDL-c) (mg/dl)

**Scrotal circumference (cm)**	0.09	0.04	0.02	0.21	0.83***
**Testis width (cm)**	0.06	0.01	0.03	0.02	0.11
**Testis length (cm)**	0.21	0.11	0.31	0.08	0.06
**Testis weight (g)**	0.07	-0.64**	0.38	0.12	0.72***
**Live body weight (kg)**	0.05	0.19	0.01	0.17	0.03


*; Significant at p<0.05, **; Significant at p<0.01 and ***; Significant at p<0.001.

Mean ± SEM of the semen characteristics and serum testosterone levels and their correlation coefficients with the serum lipid profiles are shown in tables 6 and 7. The correlation coefficients of HDLc with the number of live, normal sperm (r=0.94; p<0.001) or serum testosterone levels (r=0.88; p<0.001) was highly significant. However there were negative significant correlation coefficients between serum VLDL-c concentration and the percent of live sperm (r=-0.67; p<0.01) or serum testosterone levels (r=-0.65; p<0.01). Negative correlations were found between serum triglyceride concentration and total sperm number (r=-0.82; p<0.001), number of live, normal sperm (r=-0.55; p<0.05) or serum testosterone levels (r=-0.79; p<0.001).

**Table 6:** Mean ± standard error (SEM) of semen characteristics an d serum testosterone concentration in the Iranian male goat (n=10)


Semen volume (mL)	Sperm concentration (10^9^/mL)	Percent of live sperm (%)	Percent of abnormal sperm (%)	Total sperm number (10^9^)	Number of live sperm (10^9^)	Number of live and normal sperm (10^9^)	Serum testosterone concentration (ng/ml)

1.20 ± 0.32	2.85 ± 0.53	76.5 ± 0.34	7.5 ± 2.6	2.89 ± 0.4	1.34 ± 0.2	1.06 ± 0.8	7.3 ± 0.7


**Table 7:** Correlation coefficients between serum lipid profile with semen characteristics and serum testosterone concentration of the Iranian male goat (n=10)


Serum lipid profile/ histological measurements of testes	Total cholesterol (mg/dl)	Total triglycerides (mg/dl)	Very low density lipoprotein (VLDL-c) (mg/dl)	Low density lipoprotein (LDL-c) (mg/dl)	High density lipoprotein (HDL-c) (mg/dl)

**Semen volume (ml)**	0.13	0.27	0.17	0.42	0.21
**Sperm concentration (10^9^/ml)**	0.19	0.07	0.16	0.11	0.26
**Live sperm (%)**	0.04	0.07	-0.67**	0.03	0.29
**Abnormal sperm (%)**	0.37	0.15	0.22	0.05	0.39
**Total sperm number (10^9^)**	0.24	-0.82***	0.08	0.09	0.33
**Number of live sperm (10^9^)**	0.06	0.09	0.34	0.07	0.19
** Number of live, normal sperm (10^9^)**	0.01	-0.55*	0.14	0.26	0.94***
**Serum testosterone concentration (ng/ml)**	0.13	-0.79***	-0.65**	0.42	0.88***


*; Significant at p<0.05, **; Significant at p<0.01 and ***; Significant at p<0.001.

## Discussion

The findings demonstrated that among serum lipids, only the HDL-c levels had a positive correlation with testicular histomorphology, serum testosterone values, and seminal parameters. The steroidogenic capacity of Leydig cells in the goat’s testis and its changes during development of cellular organelles including the mitochondria and smooth endoplasmic reticulum responsible for steroid biosynthesis has been mentioned previously (22, 23). Also, it has been suggested that increased serum testosterone concentration resulted from increased Leydig cell numbers and also the amount of smooth endoplasmic reticulum within the Leydig cells (24, 25).

The mechanism(s) of beneficial effects of HDLc on testis are unknown but it seems that its cholesterol content is a major source of substrate for steroidogenic process in the Leydig cells of the testis. The main arguments for this case are as follows. In human (26), equine (8) and rodents (5), HDL-c is a major substrate source for the steroidogenic process. Sertoli cells play an important role in delivering the necessary nutrients, including lipid substrates to the sperm throughout its differentiation. In addition, despite permeation of HDL-c across the blood-testis barrier and delivery of cholesterol to Sertoli cells, LDL-c and VLDL-c particles cannot pass through this barrier, thus their access to the germ cells is excluded (27). There is evidence that HDL-c is the major plasma lipoprotein in the preruminant and ruminant (28) animals; on the other hands, the plasma levels of LDL-c and VLDL-c are minimal in this species. This phenomenon may be lead to utilize HDL-c for androgen synthesis by testicular cells.

Receptor-mediated endocytosis of HDL-c and the amount of its affinity for binding to the receptor sites have been reported by other researchers as a probable mechanism for its functions in cattle fertility (29, 30). In this regard, the high number of HDL-c receptors in the testicular tissue may be mainly related to its role in the testosterone synthesis. Padron et al. (11) have reported positive correlations between serum HDL-c levels, sperm density and viability in males.

It has been shown that normal Sertoli and Leydig cell secretory functions play a key role in the initiation and maintenance of spermatogenesis (31), maintenance of the sperm maturation process in the epididymis and stimulation of optimal sperm movements such as rapid forward progression (32). Thus, in the present study, the significant positive correlation between serum HDL-c levels with the number of live and normal sperm, testosterone profile and testis weight might be attributed to normality of the secretory function of the Sertoli and Leydig cells that resulted in normal spermatogenesis and the epididymal sperm maturation process.

The present data demonstrated a significant negative correlation in serum levels of triglycerides and VLDL-c with total sperm number, percent of live sperm, number of live and normal sperm, and serum testosterone concentration. In this regard, Ergün et al. reported that increased serum total triglyceride and VLDL-c values had deleterious effects on spermatogenesis and were significantly correlated with decreased sperm motion characteristics (13). The authors suggested that compromised fertility might be the result of a decreased testosterone concentration secondary to impaired testicular function. These findings were comparable with the present results.

Additional studies are necessary to clarify whether there is a high concentration of VLDL-c in the interstitial testicular tissue, since harmful consequences within the STs may be secondary to the secretory deficiency of Leydig cells. Alternatively other compounds induced by high serum triglyceride levels may exert a direct detrimental action outside of the seminiferous tubule (i.e. on Leydig cells).

## Conclusion

The present results seem to indicate that among serum lipids only the levels of HDL-c positively correlate with testicular parameters. The high serum triglyceride levels exert direct adverse effects at the testicular level, which are observed mainly in STs, Leydig cell characterization and semen quality. Obviously, further studies are required to elucidate the underlying mechanism(s) and to identify the sites of lipids action on the goat testis.
